# Low overpotential water oxidation at neutral pH catalyzed by a copper(ii) porphyrin†
†Electronic supplementary information (ESI) available: Fig. S1–S17, Table S1, crystallographic data in CIF format for **1**, and the X-ray crystallographic coordinates for the structure reported in this article. CCDC 1835967. For ESI and crystallographic data in CIF or other electronic format see DOI: 10.1039/c8sc04529a


**DOI:** 10.1039/c8sc04529a

**Published:** 2019-01-07

**Authors:** Yanju Liu, Yongzhen Han, Zongyao Zhang, Wei Zhang, Wenzhen Lai, Yong Wang, Rui Cao

**Affiliations:** a Department of Chemistry , Renmin University of China , Beijing 100872 , China . Email: ruicao@ruc.edu.cn; b Key Laboratory of Applied Surface and Colloid Chemistry , Ministry of Education , School of Chemistry and Chemical Engineering , Shaanxi Normal University , Xi'an 710119 , China; c State Key Laboratory for Oxo Synthesis and Selective Oxidation , Lanzhou Institute of Chemical Physics , Chinese Academy of Sciences , Lanzhou 730000 , China; d Institute of Drug Discovery Technology , Ningbo University , Ningbo 315211 , China

## Abstract

Low overpotential water oxidation under mild conditions is required for new energy conversion technologies with potential application prospects. Extensive studies on molecular catalysis have been performed to gain fundamental knowledge for the rational designing of cheap, efficient and robust catalysts. We herein report a water-soluble Cu^II^ complex of tetrakis(4-*N*-methylpyridyl)porphyrin (**1**), which catalyzes the oxygen evolution reaction (OER) in neutral aqueous solutions with small overpotentials: the onset potential of the catalytic water oxidation wave measured at current density *j* = 0.10 mA cm^–2^ is 1.13 V *versus* a normal hydrogen electrode (NHE), which corresponds to an onset overpotential of 310 mV. Constant potential electrolysis of **1** at neutral pH and at 1.30 V *versus* NHE displayed a substantial and stable current for O_2_ evolution with a faradaic efficiency of >93%. More importantly, in addition to the 4e water oxidation to O_2_ at neutral pH, **1** can catalyze the 2e water oxidation to H_2_O_2_ in acidic solutions. The produced H_2_O_2_ is detected by rotating ring–disk electrode measurements and by the sodium iodide method after bulk electrolysis at pH 3.0. This work presents an efficient and robust Cu-based catalyst for water oxidation in both neutral and acidic solutions. The observation of H_2_O_2_ during water oxidation catalysis is rare and will provide new insights into the water oxidation mechanism.

## Abstract 2

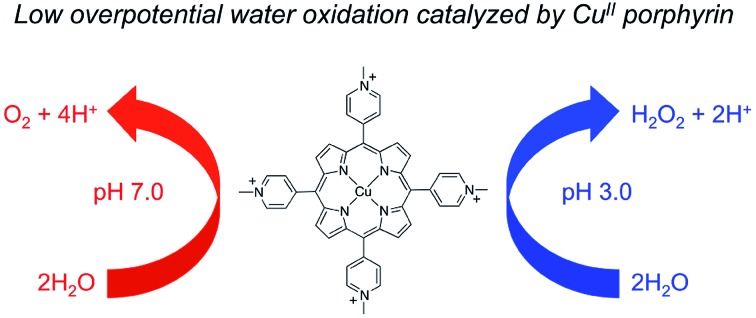
Low-overpotential water oxidation catalyzed by copper(ii) porphyrin to produce O_2_ in neutral aqueous solution and H_2_O_2_ in acidic solution.

## Introduction

The growing threat of anthropogenic climate change and the intense global energy demand on fossil fuels are among the biggest challenges faced by human beings in the current century, which makes seeking efficient carbon-neutral sources of renewable energy the most significant and urgent task of the scientific society.^1^–^3^ The global total primary energy demand will exceed 15 000 Mtoe (million tons of oil equivalent) in 2025 and reach 18 000 Mtoe in 2040. Among all the renewable energy sources, sunlight is the ideal energy resource to meet such a demand. One of the biggest challenges of sunlight utilization is the large-scale economy efficient conversion and storage.^4^–^8^ Nature, however, presents a fascinating scenario to solve this problem by using photosystem II (PSII) to store solar energy as chemical energy in a scale of sustaining the whole life circle on the earth.^9^–^11^ PSII is a membrane protein complex located in the thylakoid membranes and performs a series of light-induced electron transfer reactions leading to the splitting of water into electrons/protons and molecular dioxygen.^6^,^12^,^13^ Upon an initial light-induced charge separation, chlorophyll (P680) with a magnesium porphyrin core is converted to a cation radical species P680˙^+^, which subsequently abstracts an electron from the Mn_4_CaO_*x*_ cluster of the oxygen-evolving complex (OEC). After four such successive steps, water is oxidized at the OEC to release dioxygen, and the generated electrons/protons are used for the fixation of carbon dioxide. Therefore, water oxidation holds the central place of the natural energy-conversion scheme.

Extensive studies on artificial molecular catalysts for homogeneous water oxidation have been performed to unveil its mechanistic mysteries, particularly the pivotal O–O bond formation process.^1^,^3^,^12^,^14^ Such mechanistic information provides fundamental knowledge for the rational design of efficient and robust water oxidation catalysts. The natural Mn_4_CaO_*x*_ of the OEC encourages scientists to design biomimetic multinuclear metal clusters with redox flexibility suitable for the multi-electron water oxidation reaction. In this context, several notable metal clusters have been studied as OER catalysts, including tetramanganese,^15^,^16^ dimanganese,^17^,^18^ tetracobalt,^19^ tetraruthenium,^20^,^21^ diruthenium,^22^ and pentanuclear iron^23^ complexes. In these examples, intramolecular O–O bond formation through the coupling of two metal–oxo units is usually proposed. On the other hand, since the first report of mononuclear Ru complexes as active catalysts for water oxidation,^24^ many mononuclear metal complexes have been identified to be active OER catalysts.^25^–^52^ Unlike multinuclear systems, for mononuclear catalysts nucleophilic water attack to terminal metal–oxo/oxyl units is the likely O–O bond formation route. Despite these achievements, however, cheap catalysts functioning at neutral pH with low overpotentials for water oxidation are still under development.

As an alternative approach, metal porphyrins are promising to serve as water oxidation catalysts. The cation radical species P680˙^+^ is the most oxidizing redox cofactor known in biology with an estimated redox potential of 1.30 V *versus* NHE.^6^,^12^ It provides the initial oxidizing power for water oxidation (2H_2_O → O_2_ + 4H^+^ + 4e^–^, Δ*E* = 1.23 V *versus* NHE). The oxidizing equivalent is stored in the porphyrin macrocycle. Thus, it makes sense to design metal porphyrins to directly catalyze water oxidation, given that the metal core with the porphyrin macrocycle can provide one binding site for an aqua ligand. Bioinspired by this, we and others have reported a series of metal-porphyrin OER catalysts.^3^,^49^,^53^,^54^

**Fig. 1:**
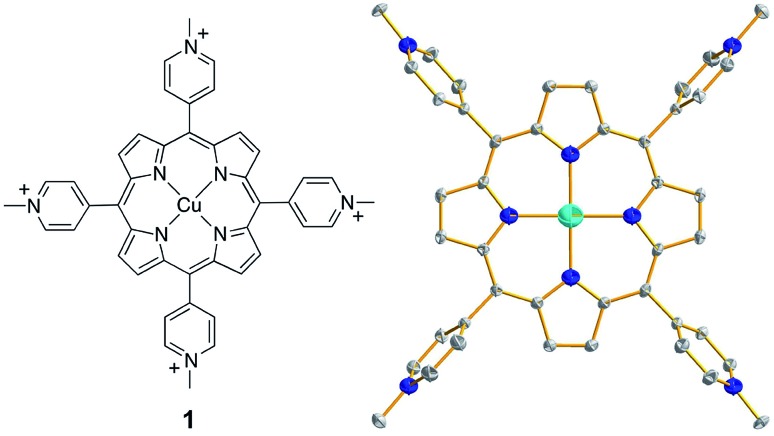
(Left) Molecular structure of **1**. (Right) Thermal ellipsoid plot of the X-ray structure of **1** (50% probability). Hydrogen atoms and counter anions are omitted for clarity.

Herein, we present a significant improvement on water oxidation catalysis with water-soluble cationic Cu^II^ porphyrin **1** (Fig. 1). Copper plays vital roles in biological and biomimetic O_2_ chemistry to mediate O–O bond formation and cleavage.^55^–^59^ As a consequence, Cu complexes have attracted increasing attention as catalysts for water oxidation.^60^–^75^ Cu^II^ porphyrin **1** can catalyze water oxidation in neutral aqueous solution with impressively small overpotentials. The onset potential of the catalytic water oxidation wave measured at the current density *j* = 0.10 mA cm^–2^ is 1.13 V *versus* NHE, which corresponds to an onset overpotential *η* = 310 mV. This value is remarkable in comparison with that of any other reported molecular OER catalysts working at neutral pH. More importantly, **1** can also catalyze the 2e water oxidation to H_2_O_2_ in acidic solutions. The selective H_2_O_2_ generation is significant because H_2_O_2_ has been extensively used as an oxidant in industry and is regarded as an alternative fuel with the benefits of easy storage and transportation.^76^–^78^ The property of **1** to catalyze both O_2_ evolution at neutral pH and H_2_O_2_ formation at acidic pH during water oxidation is very rare and will provide new insights into the catalyst design and also the mechanism for water oxidation.

## Results

### Synthesis and characterization

Complex **1** was synthesized by the reaction of Cu^II^ salts with a porphyrin ligand (see Experimental section for details). Crystals of a triflate salt were isolated by slow evaporation from a water/acetonitrile solution at room temperature, and were used for characterization and electrochemical measurements. The high-resolution mass spectrum (HRMS) of **1** showed ions at mass-to-charge ratios of 184.8086 and 518.5703, which matched the calculated values of 184.8090 for the tetravalent ion [Por-Cu]^4+^ and of 518.5700 for the divalent ion [Por-Cu-(OTf)_2_]^2+^, respectively (Fig. S1†). Importantly, the observed isotopic distributions matched those expected for these ions with separations of 0.25 and 0.50 units between each peak for tetravalent and divalent ions, respectively.

The structure of **1** was determined by single crystal X-ray diffraction. Crystallographic studies revealed that **1** crystallized in the triclinic space group *P*1[combining macron] with *Z* = 3 (crystal data and structure refinement details are summarized in Table S1†). In the X-ray structure of **1**, one molecule is located at the special position with the central Cu ion situated on the crystallographically required inversion center. The other two molecules are located at the general position and are symmetry-related through the inversion center. As a consequence, the asymmetric unit contains one and a half molecules. The Cu ion is coordinated by the porphyrin ligand through four N atoms to give a distorted square-planar geometry (Fig. 1, right). No axial ligands were found for the Cu ion in the solid state, although several co-crystallized solvent water molecules were located in the crystal lattice. Moreover, no changes were observed in the UV-vis spectrum of **1** in propylene carbonate (PC, a weak coordinating solvent) with the addition of water (Fig. S2†), further confirming that water will not bind to the Cu ion of **1**. The average Cu–N bond distance of 1.998(4) Å indicates a d^9^ Cu^II^ electronic structure. In addition, for each molecule of **1**, four CF_3_SO_3_^–^ counter anions were successfully located in the X-ray structure, further confirming the Cu^II^ oxidation state.

### Electrocatalytic water oxidation

**Fig. 2:**
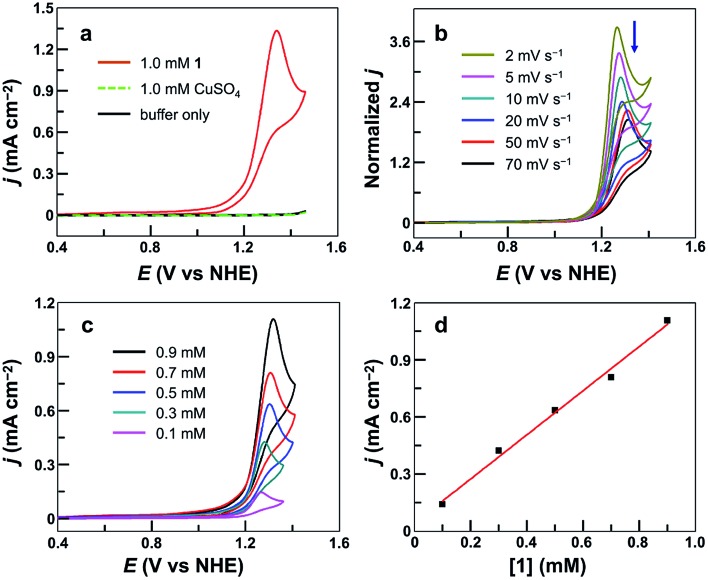
(a) CVs of **1**, CuSO_4_, and buffer-only solution at a 100 mV s^–1^ scan rate. (b) Normalized CVs of 0.50 mM **1** at different scan rates in mA cm^–2^ V^–1/2^ s^1/2^. (c) CVs of **1** at different concentrations. (d) Plot of catalytic peak current *versus* [**1**]. Conditions: FTO working electrode (*S* = 0.25 cm^2^), 0.10 M pH 7.0 phosphate buffer, and 20 °C.

The cyclic voltammogram (CV) of 1.0 mM **1** in 0.10 M pH 7.0 phosphate buffer obtained using a fluorine-doped tin oxide (FTO) working electrode displayed a pronounced catalytic wave. The onset of this catalytic wave, measured at *j* = 0.10 mA cm^–2^, is at 1.13 V *versus* NHE (all potentials reported in this work are referenced to NHE unless otherwise noted), which corresponds to an onset *η* = 310 mV (Fig. 2a). CVs of **1** in neutral H_2_O and D_2_O phosphate buffers showed a kinetic isotope effect (KIE = 1.81, Fig. S3†). The normalized catalytic currents (*i*_cat_/*ν*^1/2^) decreased with increasing scan rates (Fig. 2b), indicating that the current is associated with a catalytic process. Comparison of **1** with reported Cu-based molecular catalysts underpins its remarkable efficiency. As shown in Table 1, Cu complexes usually require strong alkaline conditions and large onset *η* values >400 mV to function as active OER catalysts. Only a few Cu catalysts are able to work at neutral pH. However, they always require large onset *η* > 600 mV to exhibit evident catalytic activities.

**Table 1:** Comparison of catalytic performances and conditions for a variety of Cu-based OER molecular catalysts

Catalyst	Catalyst concentration (mM)	pH	Onset *η*^*a*^ (mV)	TOF (s^–1^)	Reference
**Cu porphyrin** **1**	**1.0**	**7.0**	**310**	**30**	**This work**
[(bpy)Cu(OH)_2_]	1.0	12.5	540	100	60
[(TGG^4–^)Cu^II^(OH_2_)]^2–^	0.89	11	520	33	62
[(Py_3_P)Cu(OH)]^–^	0.7	8.0	400	20	67
[(dhbp)Cu(OH)_2_]	1.0	12.4	330	0.4	61
[(L2)Cu]^2–^	1.0	11.5	400	3.58	64
[(L4)Cu]^2–^	1.0	11.5	170 (not stable)	0.16	
[Cu(TMC)(H_2_O)]^2+^	1.0	7.0	600	30	73
[Cu(en)_2_]^2+^	1.0	8.0	530	0.4	72
Cu(pyalk)_2_	1.0	13.3	460	0.7	66
[pdca–Cu^II^–CO_3_H]^–^	1.0	10.0	650	20.1	69
[Cu_2_(BPMAN)(μ-OH)]^3+^	1.0	7.0	800	0.6	63
[(TMPA–Cu^II^)_2_–(μ-OH)_2_]^2+^	1.0	12.5	720	33	68

^*a*^The onset overpotential values for water oxidation mediated by molecular catalysts are generally reported and are used for comparison in the literature. Abbreviations used in this table: bpy, 2,2′-bipyridine; TGG, triglycylglycine; Py_3_P, *N*,*N*-bis(2-(2-pyridyl)ethyl)pyridine-2,6-dicarboxamidate; dhbp, 6,6′-dihydroxy-2,2′-bipyridine; L2 and L4, *N*_1_,*N*_1_′-(1,2-phenylene)bis(*N*_2_-methyloxalamide) derivatives; TMC, 1,4,8,11-tetramethyl-1,4,8,11-tetraazacyclotetradecane; en, 1,2-ethylenediamine; pyalk, 2-pyridyl-2-propanoate; pdca, *N*,*N*′-2,6-dimethylphenyl-2,6-pyridinedicarboxamidate; BPMAN, 2,7-[bis(2-pyridylmethyl)-aminomethyl]-1,8-naphthyridine; TMPA, bis((6-methyl-2-pyridyl)methyl)(2-pyridylmethyl)amine.

The molecular nature of **1** for water oxidation was verified. No catalytic currents were observed up to 1.45 V in a buffer-only solution and in a 1.0 mM CuSO_4_ solution under identical conditions (Fig. 2a). The catalytic currents increased with increasing concentration of **1** (Fig. 2c), exhibiting a linear dependence (Fig. 2d). Further analysis of the catalytic water oxidation wave by using foot of the wave analysis (FOWA,^64^,^79^–^83^ Fig. S4†) showed that the observed reaction rate constant remained almost unchanged at different concentrations of **1**. These results together indicate the first-order dependence of the catalytic reaction kinetics on **1**. In addition, the coordination-caused inhibition by either phosphate anions (Fig. S5†) or acetonitrile molecules (Fig. S6†) is strong evidence for Cu-centered homogeneous catalysis.

**Fig. 3:**
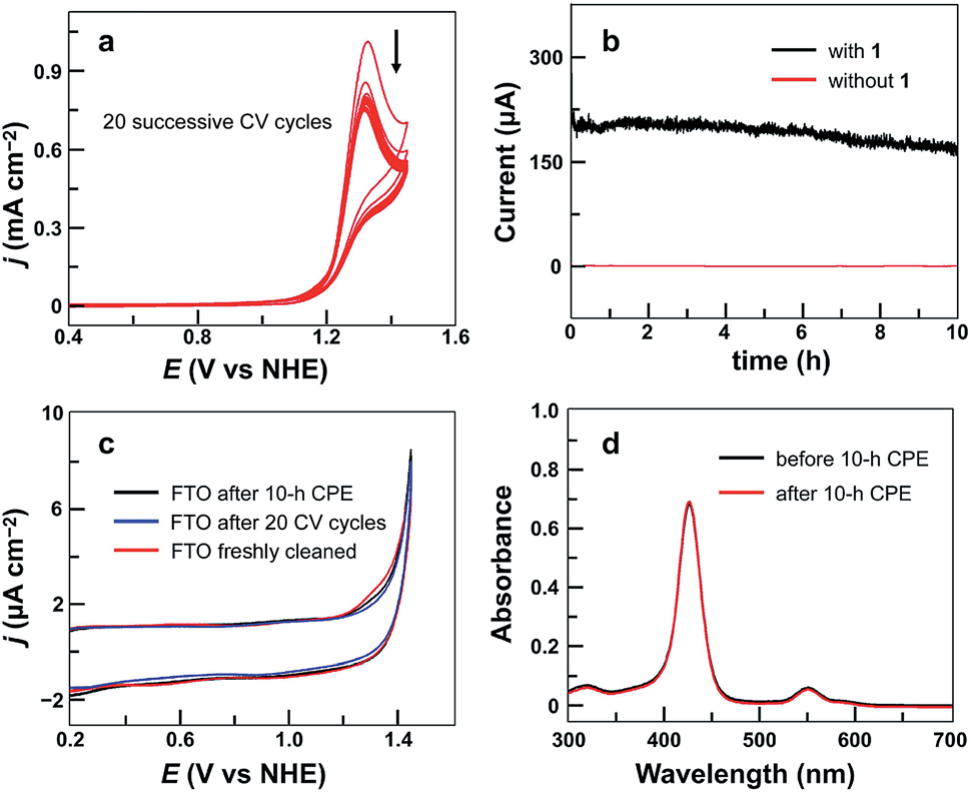
(a) 20 successive CV cycles of 0.75 mM **1** with the FTO working electrode (*S* = 0.25 cm^2^) at a 50 mV s^–1^ scan rate. (b) Catalytic currents in CPE with or without **1** (0.75 mM) using the FTO electrode (*S* = 1.0 cm^2^) at an applied potential of 1.30 V. (c) CVs of the FTO electrode after 10 h CPE with **1**, the FTO electrode after 20 successive CV cycles with **1**, and a freshly cleaned FTO electrode in the buffer-only solution. Conditions: 0.10 M pH 7.0 phosphate buffer, 100 mV s^–1^ scan rate, and 20 °C. (d) UV-vis spectra of **1** before and after 10 h CPE.

The stability of **1** under catalytic conditions was evaluated. Successive CV scans of **1** showed no current increase (Fig. 3a). The peak current slightly decreased in the first several scans and then remained almost constant. The initial small drop of the peak current is due to the generation and accumulation of O_2_ gas bubbles on the surface of the FTO working electrode. Constant potential electrolysis (CPE) of **1** for 10 h at 1.30 V displayed a substantial and stable current (Fig. 3b). The FTO working electrodes after 20 CV scans and after CPE displayed no catalytic current in freshly prepared buffer-only solutions (Fig. 3c). Surface analysis of the electrode after CPE by scanning electron microscopy (SEM) and energy dispersive X-ray spectroscopy (EDX) showed no sign of the deposition of any heterogeneous Cu-based materials (Fig. S7†). In addition, the solution after CPE was analyzed by UV-vis spectroscopy, showing a negligible change as compared to that before CPE (Fig. 3d). These results strongly supported the molecular nature of **1** and its stability under catalytic OER conditions.

The evolved O_2_ during CPE was then determined, revealing a faradaic efficiency of >93% for O_2_ evolution (Fig. S8†). The turnover number was calculated to be 5.0 based on all catalyst molecules in the solution. A diffusion-controlled Cu^II^/Cu^I^ redox couple of **1** was found at *E*_1/2_ = 0.06 V (Fig. S9†). With this information, the plot of *i*_cat_/*i*_d_*versus ν*^–1/2^ shows a linear relationship (Fig. S10†), which is consistent with a pure kinetic behavior in this scan rate range. A turnover frequency (TOF) value of 30 s^–1^ can be calculated using established methods.^84^ This value is remarkable because it is obtained at neutral pH and is comparable to the values presented by those Cu-based molecular catalysts working in alkaline solutions. Moreover, this TOF value with **1** is obtained at much smaller overpotentials as compared to that of reported Cu-based catalysts (Table 1).

### pH-Dependent electrochemical studies

**Fig. 4:**
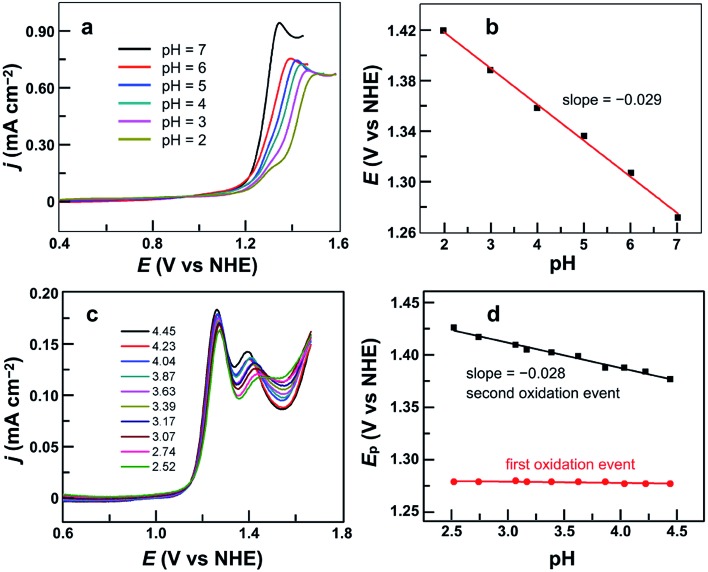
(a) LSV curves of 0.50 mM **1** in 0.10 M pH 2.0–7.0 phosphate buffers. (b) Plot of potential measured at *j* = 0.45 mA cm^–2^*versus* pH. (c) DPV curves of 0.50 mM **1** in 0.10 M pH 2.52–4.45 phosphate buffers. (d) Plots of the first and second oxidation peak potentials *versus* pH from 2.52 to 4.45. Conditions: GC working electrode, scan rate 100 mV s^–1^, and 20 °C.

After establishing the molecular nature of **1** for catalysis, we next carried out pH-dependent studies. Fig. 4a displayed the linear sweep voltammetry (LSV) curves of 0.50 mM **1** in 0.10 M pH 2.0–7.0 phosphate buffers obtained using glassy carbon (GC) electrodes. Significantly, **1** was active even in strong acidic solutions (*i.e.*, pH < 3). This result is additional support for the molecular nature of **1** as the real water oxidation catalyst because any Cu oxides that may possibly be generated upon the decomposition of **1** are unstable in acidic solutions. The plot of the potential as measured at *j* = 0.45 mA cm^–2^*versus* pH gives a linear relationship (Fig. 4b). The slope is –0.029 V per pH unit, indicating that the catalytic cycle involves an unusual 2e^–^/1H^+^ redox step. Notably, in strong acidic solutions at pH 2.0–3.0, a shoulder peak at 1.29 V prior to the catalytic water oxidation wave can be identified, which is assigned to the 1e oxidation of **1**.

On the basis of these results, we studied the pH-dependent electrochemistry by using the differential pulse voltammetry (DPV) method. The DPV results of **1** displayed two oxidation events in 0.1 M pH 2.52–4.45 phosphate buffers (Fig. 4c). At pH 2.52, these two oxidation events are at *E*_p,a_ = 1.27 and 1.44 V, respectively. Significantly, the first oxidation is pH-independent, while the second one is pH-dependent. The plots of the first and second oxidation peak potentials *versus* pH values are depicted in Fig. 4d, showing that the *E*_p,a_ values of the first oxidation remain nearly identical but those of the second oxidation show a monotonic decrease with a slope of –0.028 V per pH unit. This number is the same as that obtained in the abovementioned LSV studies, suggesting that the second oxidation corresponds to the 2e^–^/1H^+^ process. With the further increase of the solution pH, the second oxidation wave steadily moves towards the first oxidation wave (Fig. S11†), and they eventually become superimposed under near neutral conditions. Notably, in the literature, overpotentials at the initial foot of the DPV oxidation wave and at the half-peak position of the catalytic CV oxidation wave are also reported. In the case of **1** at pH 7.0, the potential at the initial foot of the DPV oxidation wave is 1.12 V, while the half-peak potential of the catalytic CV oxidation wave is 1.26 V. These give *η* = 300 mV (at the initial foot of DPV) and 440 mV (at the half-peak position of the CV wave) for catalytic water oxidation, respectively. The overpotential values determined from the initial foot of both the CV and DPV oxidation waves of **1** are identical in our experiments.

**Fig. 5:**
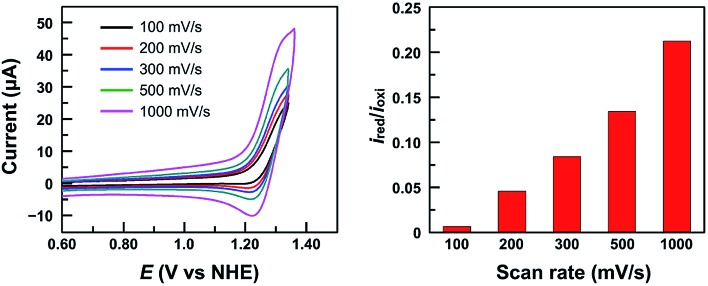
CVs of 1.0 mM **1** in 0.10 M pH 2.52 phosphate buffer by reversing the scan at 1.35 V with different scan rates (Left) and the ratio of the reduction peak current *versus* the oxidation peak current *i*_red_/*i*_oxi_ (Right). Conditions: GC working electrode and 20 °C.

It is worth noting that at pH 2.52, the current of the second oxidation is much smaller than that of the first oxidation in the DPV plot of **1** (Fig. 4c). With increasing pH, the second oxidation moves towards the first oxidation, and its current also increases significantly. This trend implies a pH-dependent chemical reaction/transformation of the 1e-oxidized species of **1**. In order to get more insights into the first oxidation process, we measured the CV of **1** at pH 2.52 by reversing the scan prior to the catalytic water oxidation wave (*i.e.*, <1.35 V). As shown in Fig. 5, almost no reduction peak can be observed at a 100 mV s^–1^ scan rate. However, a small but significant reduction peak appears at a 200 mV s^–1^ scan rate and the current increases with the scan rate. Moreover, the ratio of the reduction peak current *versus* the oxidation peak current increases with the scan rate (Fig. 5). This result further supports the chemical reaction/transformation of the 1e-oxidized species of **1**, leading to its corresponding reduction wave only being detectable on reversing the scan with large scan rates.

Next, we performed electrolysis of **1** at 1.40 V in a pH 3.0 phosphate buffer solution. Thin-layer spectroelectrochemical analysis showed an absorbance decrease at 425 and 550 nm but the absorbance increases in the 600–800 nm range (Fig. S12†). These results together suggest that the first oxidation of **1** is ligand-centered to form a Cu^II^ porphyrin cation radical species. In addition, we can oxidize **1** in a PC solution using ceric ammonium nitrate (CAN) as the chemical oxidant. As shown in Fig. S13,† similar changes were observed in this chemical oxidation process: the absorbance decreases at 544 nm but increases in the 590–800 nm range, which is characteristic for the ligand-centered oxidation to give porphyrin cation radical species.

Interestingly, the electrochemical behaviors of this first oxidation event of **1** are different in aqueous solutions *versus* those in organic solutions. In aqueous solutions, the first oxidation wave is irreversible at a 100 mV s^–1^ scan rate. However, in organic solutions, complex **1** displays a quasi-reversible oxidation process even at slow scan rates (*i.e.*, 30 mV s^–1^, Fig. S14†). We propose that upon the first oxidation, the 1e-oxidized species of **1** can bind an aqua ligand at the axial position of the Cu ion, which can then initiate the water oxidation process. This chemical reaction/transformation is unlikely to happen for the 1e-oxidized species of **1** in the organic PC solution.

### H_2_O_2_-related electrochemical studies

Significantly, during the electrolysis of **1** in pH 3.0 buffer solution with applied potentials <1.40 V, H_2_O_2_ was generated and was detected by the addition of sodium iodide (Fig. S15†). In order to determine the faradaic efficiency of H_2_O_2_ generation, we used a rotating ring (Pt)–disk (GC) electrode (RRDE) with CPE of the GC disk electrode at 1.40 V and CPE of the Pt ring electrode at 0.70 V in a pH 3.0 phosphate buffer solution. Stable currents were observed on both electrodes (Fig. S16†), giving a faradaic efficiency of 45%. This value is much smaller than 100%, and is likely caused by some level of oxidation of H_2_O_2_ at the disk electrode upon its formation. This provides strong evidence for the electrocatalytic features of **1** to catalyze 2e water oxidation to generate H_2_O_2_ in acidic solutions.

**Fig. 6:**
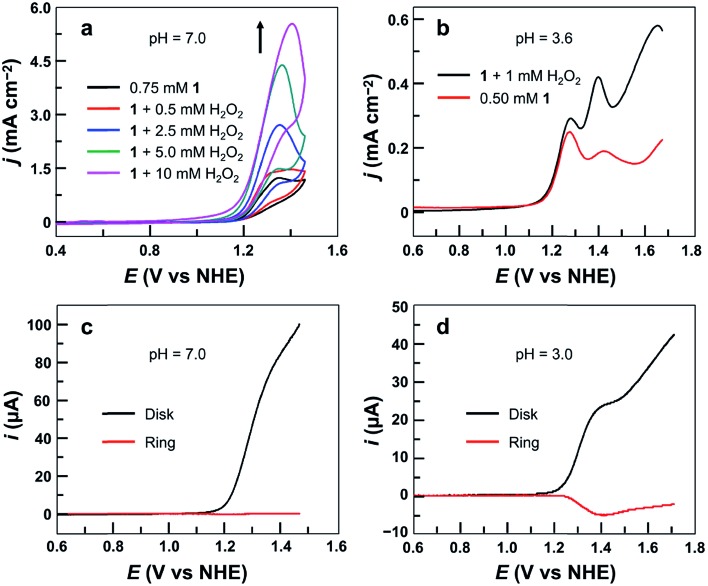
(a) CVs of 0.75 mM **1** with the addition of H_2_O_2_ using the GC electrode at a 100 mV s^–1^ scan rate. (b) DPV curves of 0.50 mM **1** with or without 1.0 mM H_2_O_2_. Conditions: 0.10 M phosphate buffer and 20 °C. (c) RRDE analysis of 0.5 mM **1** in 0.10 M pH 7.0 phosphate buffer. (d) RRDE analysis of 0.50 mM **1** in 0.10 M pH 3.0 phosphate buffer. The potential of the ring electrode is maintained at 0.70 V. Rotation rate is 1000 rpm, and scan rate is 10 mV s^–1^.

Moreover, the CVs of **1** with addition of authentic H_2_O_2_ show remarkably increased catalytic waves (Fig. 6a). As a control, the CV of H_2_O_2_ under the same conditions without **1** showed a negligible current (Fig. S17†). We next studied the DPV of **1** with H_2_O_2_ in 0.10 M pH 3.6 phosphate buffer (Fig. 6b). Significantly, with the addition of H_2_O_2_, the first oxidation wave remained almost unchanged, but the second oxidation wave became much more pronounced, confirming that the second oxidation was associated with the H_2_O_2_-related species.

More importantly, the association of the second oxidation in the DPV of **1** with H_2_O_2_-related species can be further verified by using RRDE analysis. Electrochemical studies of **1** were conducted in 0.10 M pH 7.0 or pH 3.0 phosphate buffer solutions using LSV of the GC disk electrode with a scan rate of 10 mV s^–1^ and CPE of the Pt ring electrode at 0.70 V. At pH 7.0, no anodic current of H_2_O_2_ oxidation could be detected at the Pt ring electrode (Fig. 6c). However, in the pH 3.0 solution, a large current due to H_2_O_2_ oxidation was observed at the Pt ring electrode (Fig. 6d). This difference is likely caused by the different potentials required for the second oxidation in pH 7.0 and pH 3.0 solutions. At neutral pH, the second oxidation wave is superimposed with the first oxidation wave of **1** (Fig. S11†). As a consequence, this H_2_O_2_-related species is instantly oxidized on the GC disk electrode upon its formation. At pH 3.0, however, more positive potentials (*i.e.*, >1.40 V) are required for the oxidation of this H_2_O_2_-related species (Fig. 4c). Therefore, it can be detected at the Pt ring electrode. Importantly, as shown in Fig. 6d, along with the generation and accumulation of the H_2_O_2_-related species in LSV, the current at the Pt ring electrode constantly increases. However, at more positive potentials (*i.e.*, >1.40 V), the current at the Pt ring electrode starts to decrease. This phenomenon is consistent with the oxidation/degradation of the H_2_O_2_-related species at the GC disk electrode at more positive potentials. The synchronization between the generation/degradation of the peroxide species at the disk electrode and the increase/decrease of the current at the ring electrode during RRDE measurements is supportive of (1) the formation of H_2_O_2_ during water oxidation with **1** in acidic media and (2) the association of the second oxidation in the DPV of **1** with H_2_O_2_-related species.

## Discussion

Since the first report by Mayer and co-workers in 2012, Cu-based molecular OER catalysts have grown rapidly due to the advantages of high earth abundance, low cost and rich redox features of copper. On the other hand, copper enzymes play significant roles in numerous O_2_ activation processes in nature, and biomimetic Cu complexes have also been extensively investigated for such O_2_ chemistry. These features make Cu complexes the most attractive candidates as OER catalysts. For the reported mononuclear Cu complexes, however, their catalytic activity is mostly limited to functioning at high overpotentials and in alkaline solutions. A water nucleophilic attack (WNA) to high-valent Cu^IV^–oxo or Cu^III^–oxyl units is commonly suggested for the O–O bond formation mechanism. Electrochemical evidence was provided by Meyer and co-workers to support the presence of the nascent Cu^II^–OOH species. Very recently, Maseras, Llobet and co-workers proposed an alternative single electron transfer–water nucleophilic attack (SET–WNA) mechanism. In this mechanism, a formal Cu^IV^–OH species was proposed as the active species to react with a hydroxyl ion for O–O bond formation. Instead of transferring two electrons in a direct WNA step, two single electron transfer steps from a hydroxyl ion to Cu^IV^–OH take place with the involvement of an (HO···OH)^–^ moiety. This mechanism avoids the involvement of high-energy Cu^IV^–oxo, but alkaline conditions are still required. Kieber-Emmons and co-workers recently described a dinuclear Cu complex [Cu^II^_2_–(μ-OH)_2_]^2+^, which catalyzed water oxidation with an onset *η* = 720 mV at pH = 12.5. The 2e-oxidized form [Cu^III^_2_–(μ-O)_2_]^2+^ was considered to couple the two bridging oxo atoms to form a “side-on” μ-(η^2^:η^2^)-peroxo dinuclear Cu^II^ intermediate. Subsequent redox isomerization converts this “side-on” structure to an “end-on” μ-(1,2)-peroxo structure. However, the authors mentioned that WNA to one oxo group of [Cu^III^_2_–(μ-O)_2_]^2+^ was also possible for O–O bond formation. Liao, Zhang and co-workers described another dinuclear Cu complex [Cu^II^_2_–(μ-OH)]^3+^, which catalyzed water oxidation at neutral pH with an onset *η* = 800 mV. After 2e oxidation, the oxygen atom of the terminal Cu^III^–OH unit coupled with the bridging oxo atom of [Cu^III^_2_–(μ-O)]^4+^ to form the O–O bond. For these dinuclear Cu complexes, although high-energy Cu^IV^ (*i.e.*, Cu^IV^–oxo) active species were avoided in the proposed mechanisms, dinuclear forms are intrinsically more difficult to be oxidized than mononuclear forms because of their larger overall positive charges. As a consequence, these dinuclear Cu catalysts still worked at high overpotentials.

In the present article, we report on a water-soluble cationic mononuclear Cu^II^ porphyrin **1** as a highly efficient and stable molecular electrocatalyst for water oxidation. Complex **1** can catalyze the 4e water oxidation to generate O_2_ in neutral aqueous solution with a small onset *η* = 310 mV. This value is remarkable in comparison with that with any other reported molecular OER catalysts working at neutral pH. More importantly, **1** can also catalyze the 2e water oxidation to produce H_2_O_2_ in acidic solutions. The impressively small overpotentials for water oxidation, and the features of both O_2_ evolution at neutral pH and H_2_O_2_ formation at acidic pH during water oxidation catalysis, are unique for complex **1** as a water oxidation catalyst.

Although the complete catalytic cycle of water oxidation with **1** is difficult to be fully understood based on the current experimental results, we can observe H_2_O_2_ generation during water oxidation with **1** in acidic solutions and can also provide strong evidence to support the fact that the second oxidation of **1** is associated with H_2_O_2_-related species. This result suggests that the 1e-oxidized species of **1** triggers O–O bond formation. This proposal may explain why complex **1** can catalyze water oxidation in neutral aqueous solutions with small overpotentials. For example, one can imagine that the initiation of O–O bond formation through 1e-oxidized species, instead of involving high-valent Cu^IV^–oxo/Cu^III^–oxyl species, should certainly decrease the potentials required for water oxidation. More mechanistic studies are in progress with aims to better understand the catalytic cycle, in particular, O–O bond formation, of water oxidation with this Cu^II^ porphyrin catalyst.

## Experimental section

### General materials and methods

All reagents were purchased from commercial suppliers and used as received unless otherwise noted. Deionized water was used in all experiments. Tetrakis(4-*N*-methylpyridyl)porphyrin was prepared according to modified literature procedures.^49^ High-resolution mass spectrometric measurements were made on a Brüker Fourier transform ion cyclotron resonance mass spectrometer APEX IV. Electronic absorption spectra were recorded on a Cary 50 spectrophotometer. The morphologies of the FTO surface before and after electrolysis were examined using a JSM-6700F field emission scanning electron microscope (FE-SEM). The EDX spectra were collected from three randomly selected areas of each sample. In addition, the materials were analyzed at several local spots to ensure their chemical homogeneity. Images were obtained with an acceleration voltage of 5 kV.

### Synthesis of [Por-Cu](OTf)_4_ (**1**)

Tetrakis(4-*N*-methylpyridyl)porphyrin tetratosylate (0.68 g, 0.50 mmol) and CuCl_2_ (0.47 g, 3.5 mmol) were added into H_2_O (50 mL). The mixture solution was then refluxed for 5 h in the dark. After cooling to room temperature, the reaction solution was precipitated by adding it to a saturated aqueous NH_4_PF_6_ solution under stirring. The precipitate that formed was filtered off, washed with water, and dried in a vacuum to give red crystalline solids of [Por-Cu](PF_6_)_4_ (0.53 g, 0.40 mmol, yield 80%). These red solids were added into a mixture of CH_3_CN/H_2_O (1 : 1), and the PF_6_^–^ anions were exchanged by CF_3_SO_3_^–^ anions on an anion exchange resin by slow stirring at room temperature for three days. The solution was filtered, and the resin was washed with CH_3_CN. The solvents were then removed under reduced pressure to give red crystalline solids. Red needle-like crystals of **1** were obtained by slow evaporation from CH_3_CN and H_2_O at room temperature with a yield of 85% (0.45 g, 0.35 mmol). HRMS: calcd for the tetravalent ion [Por-Cu]^4+^, 184.8090; found, 184.8086; calcd for the divalent ion [Por-Cu-(OTf)_2_]^2+^ 518.5700; found, 518.5703 (Fig. S1†). Anal. calcd for [Cu(C_44_H_36_N_8_)](CF_3_SO_3_)_4_: C, 43.13; H, 2.72; N, 8.38. Found: C, 43.32; H, 2.81; N, 8.50.

### Crystallographic studies

The complete dataset for **1** (CCDC ; 1835967 †) was collected. A single crystal suitable for X-ray analysis was coated with Paratone-N oil, suspended in a small fiber loop, and placed on a Bruker D8 Venture X-ray diffractometer at 150(2) K. Diffraction intensities were measured using graphite monochromated Mo Kα radiation (*λ* = 0.71073 Å). Data collection, indexing, data reduction, and final unit cell refinements were carried out using APEX2;^85^ absorption corrections were applied using the program SADABS.^86^ The structure was solved by direct methods using SHELXS^87^ and refined against *F*^2^ on all data by full-matrix least-squares using SHELXL,^88^ following established refinement strategies. In the single crystal X-ray structure of **1**, all non-hydrogen atoms were refined anisotropically. All hydrogen atoms binding to carbon were included in the model at geometrically calculated positions and refined using a riding model. The isotropic displacement parameters of all hydrogen atoms were fixed to 1.2 times the *U* value of the atoms they are linked to (1.5 times for methyl groups). Details of the data quality and a summary of the residual values of the refinements are listed in ESI Table S1.†

### Electrochemical studies

All electrochemical experiments were carried out using a CH Instruments electrochemical analyzer (model CHI660E) at 20 °C unless otherwise noted. A three-compartment cell was used with a 0.25 cm^2^ FTO or a 0.07 cm^2^ GC electrode as the working electrode, saturated Ag/AgCl as the reference electrode, and a graphite rod as the auxiliary electrode. All potentials are reported *versus* the NHE through the addition of 0.199 V to the measured potential. The working electrode was ultrasonically washed with deionized water. Electrolysis was recorded in 0.10 M pH 7.0 phosphate buffer containing 0.75 mM **1** at 1.30 V in a three-compartment cell with a 1.0 cm^2^ FTO working electrode, a graphite rod auxiliary electrode, and an Ag/AgCl reference electrode. The produced O_2_ was analyzed using an Ocean Optics FOXY probe (Model NeoFox). The generated H_2_O_2_ was detected by electrochemical measurements using RRDE analysis. Electrochemical studies of **1** were conducted in 0.10 M pH 7.0 or pH 3.0 phosphate buffer using LSV of the disk electrode with a scan rate of 10 mV s^–1^ and electrolysis of the ring electrode at 0.70 V.

## Conflicts of interest

The authors declare no competing financial interests.

## Supplementary Material

Supplementary information

Crystal structure data
