# Supplementary material for: Low overpotential water oxidation at neutral pH catalyzed by a copper(ii) porphyrin
Source: Chem Sci. 2019 Jan 7;10(9):2613–22. doi: 10.1039/c8sc04529a (PMC6419937; doi:10.1039/c8sc04529a)
Supplement: Supplementary file 1 [file SC-010-C8SC04529A-s001.pdf]

Electronic Supplementary Material (ESI) for Chemical Science.
This journal is © The Royal Society of Chemistry 2019

Electronic Supplementary Information

Low overpotential water oxidation at neutral pH catalyzed by a copper(II) porphyrin

Yanju Liu,[1] Yongzhen Han,[1] Zongyao Zhang,[1] Wei Zhang,[2] Wenzhen Lai,[1] Yong Wang,[3,4] and Rui Cao[1,2,*]

[1]Department of Chemistry, Renmin University of China, Beijing 100872, China

[2]Key Laboratory of Applied Surface and Colloid Chemistry, Ministry of Education, School of Chemistry and Chemical Engineering, Shaanxi Normal University, Xi'an 710119, China

[3]State Key Laboratory for Oxo Synthesis and Selective Oxidation, Lanzhou Institute of Chemical Physics, Chinese Academy of Sciences, Lanzhou 730000, China

[4]Institute of Drug Discovery Technology, Ningbo University, Ningbo 315211, China

Correspondence E-mail: ruicao@ruc.edu.cn

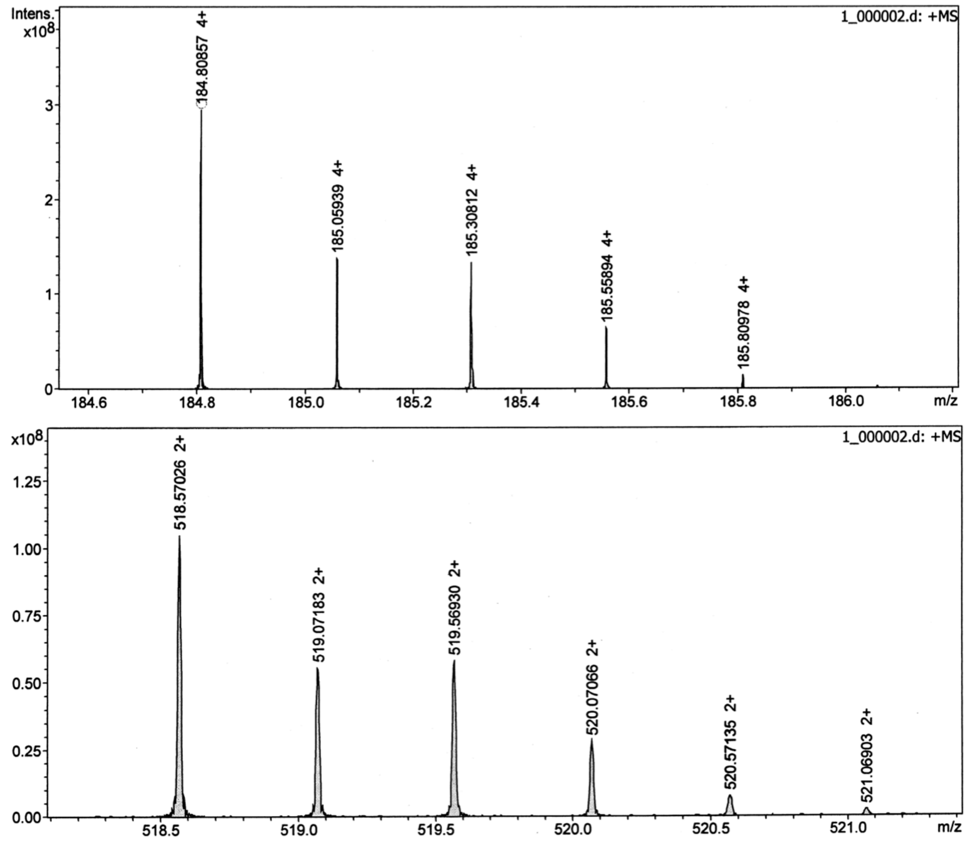

Figure S1. HRMS of complex **1**. (Top) The ion at a mass-to-charge ratio of 184.8086 matches the calculated value of 184.8090 for the tetravalent ion $[Cu\text{-}TMPyP]^{4+}$. (Bottom) The ion at a mass-to-charge ratio of 518.5703 matches the calculated value of 518.5700 for the divalent ion $[Cu\text{-}TMPyP\text{-}(OTf)_2]^{2+}$.

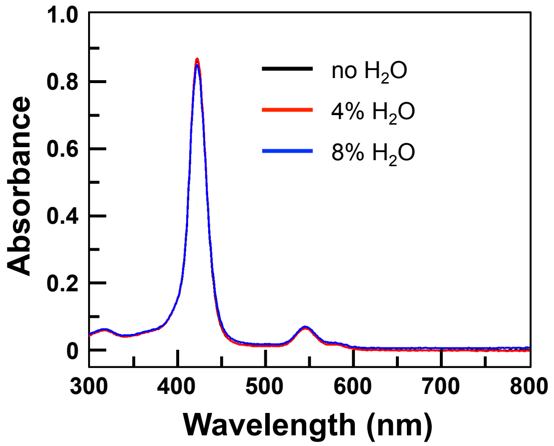

Figure S2. UV-vis spectra of **1** in propylene carbonate (PC) solutions without or with addition of 4% and 8% H_2O, showing no changes. This result suggests no axial aqua binding on Cu^{II} center of complex **1**.

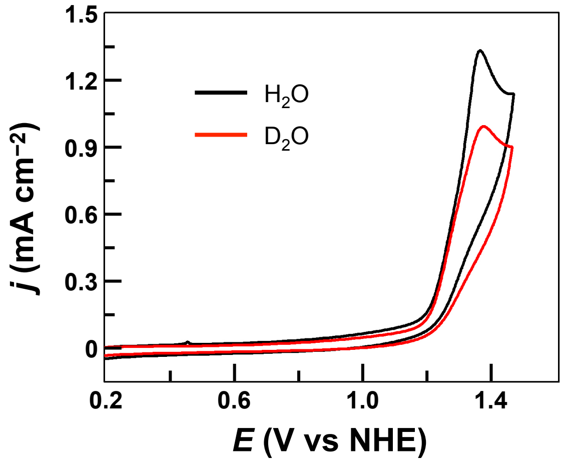

Figure S3. CVs of 0.75 mM **1** in H_2O and D_2O. Conditions: 0.10 M neutral phosphate buffer, GC working electrode, scan rate 100 mV s^{-1}, 20 °C.

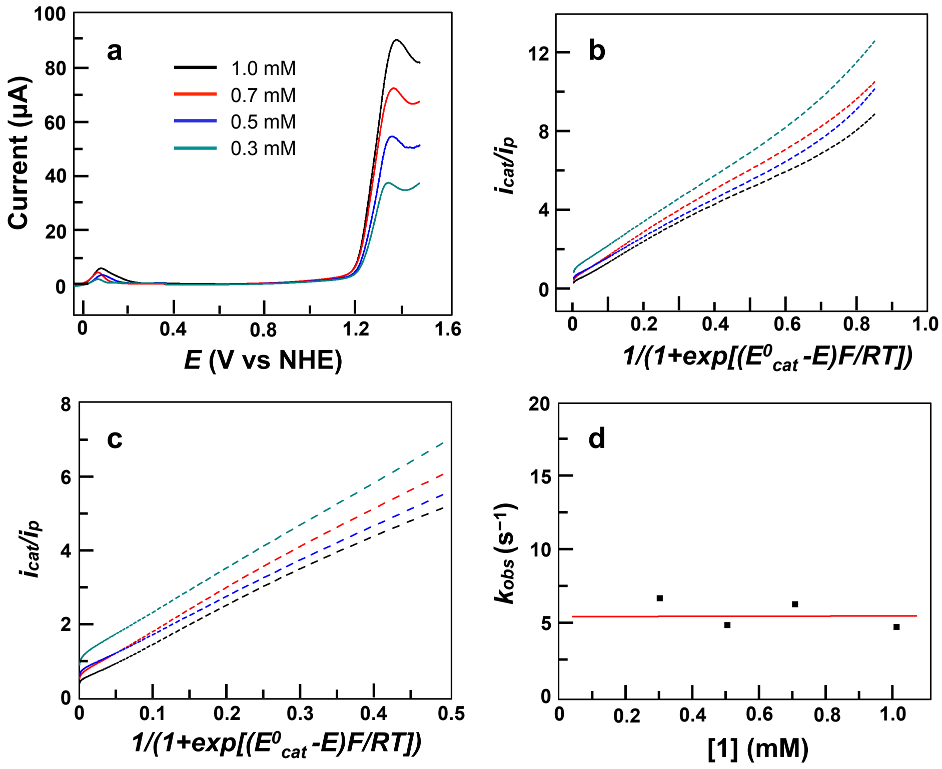

Figure S4. (a) CVs of **1** at different concentrations. Conditions: 0.10 M pH 7.0 phosphate buffer, GC working electrode, scan rate 100 mV s^{-1}, 20 °C. (b, c) FOWA plotting i_{cat}/i_p versus 1/(1+exp[(E^0_{cat}-E)F/RT]) at each concentration (as the same color above). (d) Plot of reaction kinetics (k_{obs}) extracted from FOWA when E^0_{cat} = 1.25 V is used. The red line represents the kinetics as function of catalyst concentration. If E^0_{cat} = 1.16 V is used, k_{obs} = 28 s^{-1} is obtained from the analysis.

FOWA is applied by using the following equation:

$$\frac{i_{cat}}{i_p} = \frac{2.24n\sqrt{\frac{RT}{F\upsilon}k_{obs}}}{1+\exp\left[\frac{F}{RT}(E^0_{cat}-E)\right]}$$

in which E^0_{cat} is the standard potential for the catalysis-initiating redox couple (1.25 V calculated from DPV), i_{cat} is the current intensity in the presence of substrate, i_p is the current intensity of non-catalytic process (we approximate this current to the current associated with the Cu^{II}/Cu^{I} couple here), n is the number of electrons involved in the catalytic cycle (4e in water oxidation), F is the faraday constant, v is the scan rate, k_{obs} is defined as "$k_{cat} \cdot C^0_A$" where C^0_A is the concentration of the substrate (water), and R is 8.314 J mol^{-1} K^{-1}, T is 293 K. CVs of catalyst **1** at different concentration are shown in Figure S4a. Now, k_{obs} can be extracted from the plot of i_{cat}/i_p versus $1/(1+\exp[(E^0_{cat}-E)F/RT])$.

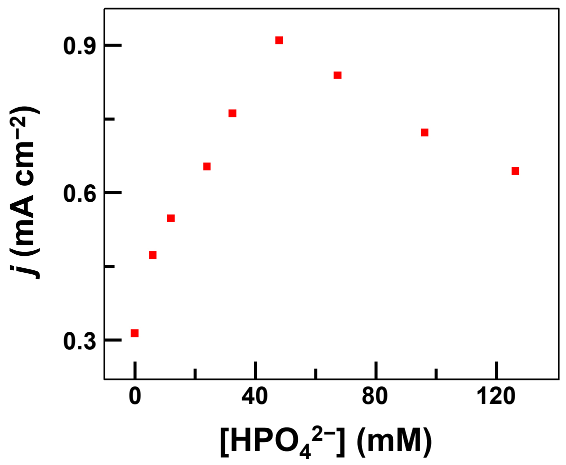

Figure S5. Plot of catalytic peak current versus [HPO_4^{2-}]. Conditions: 0.50 mM catalyst **1**, GC working electrode, pH = 7.0, scan rate 100 mV s^{-1}, 20 °C. The ionic strength was maintained as constant during these measurements with addition of sodium sulfate.

The activity of **1** displayed a volcano-type dependence on phosphate concentrations. At low phosphate concentrations, the catalytic peak current increased rapidly with [HPO_4^{2-}] up to 48 mM. Further increase of phosphate concentrations caused the decrease of catalytic currents. These results suggest that the phosphate buffer anion play several roles in the OER catalysis by (1) assisting the proton transfer process and (2) binding to the Cu center to prevent the coordination and thus activation of water molecules.

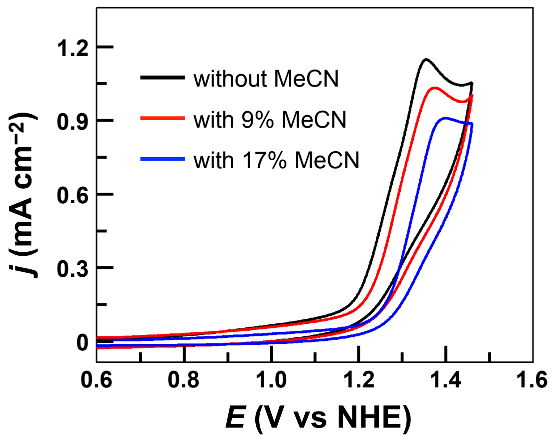

Figure S6. CVs of 0.50 mM **1** in 0.10 M pH 7.0 phosphate buffer with addition of different amounts of acetonitrile. Conditions: GC working electrode, scan rate 100 mV s^{-1}, 20 °C.

This result shows that addition of acetonitrile also caused apparent inhibition, confirming the competitive coordination on the Cu ion.

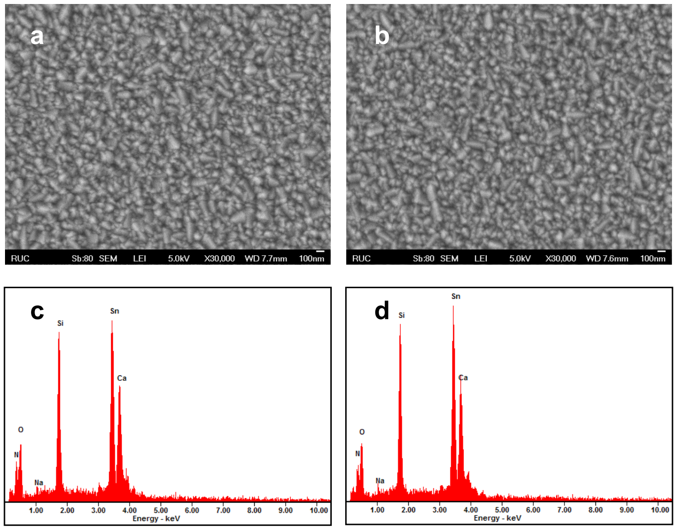

Figure S7. SEM images and EDX spectra of the FTO working electrode before (a, c) and after (b, d) 10-h CPE.

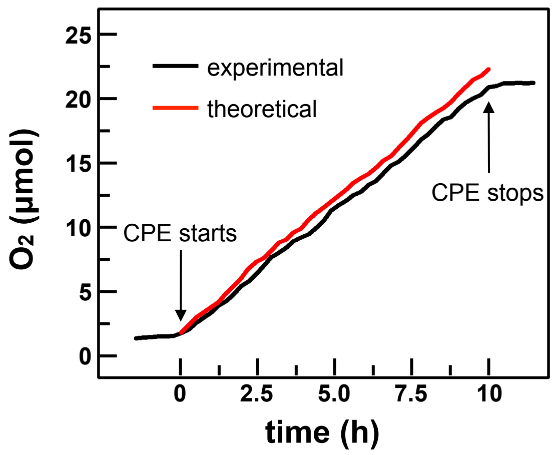

Figure S8. Observed and theoretical amounts of O_2 evolved during CPE with **1**. Conditions: 0.1 M pH 7.0 phosphate buffer, FTO working electrode (S = 1.0 cm^2), applied potential 1.30 V, 20 ºC.

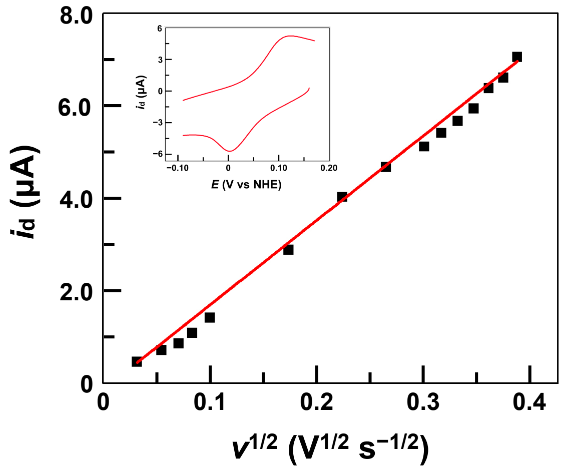

Figure S9. Dependence of the Cu^{II}/Cu^{I} redox potential of **1** on the square root of scan rates. The oxidation peak currents vary linearly with the square root of scan rates, indicating a diffusion-controlled process. Conditions: argon atmosphere, 0.50 mM **1**, 0.1 M pH 7.0 phosphate buffer, GC working electrode, 20 ºC. Inset: representative Cu^{II}/Cu^{I} redox couple of **1**.

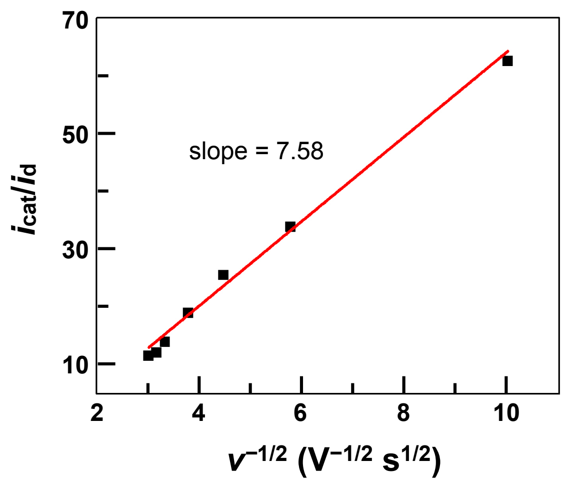

Figure S10. Plot of i_{cat}/i_d versus $\nu^{-1/2}$ with a slope of 7.58. Conditions: 0.50 mM **1**, 0.1 M pH 7.0 phosphate buffer, GC working electrode, 20 °C.

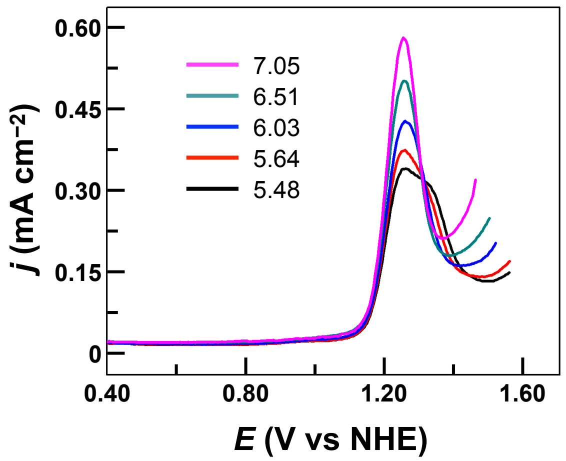

Figure S11. DPVs of 0.50 mM **1** in 0.10 M pH 5.48-7.05 phosphate buffers. Conditions: GC working electrode, scan rate 100 mV s^{-1}, 20 ºC.

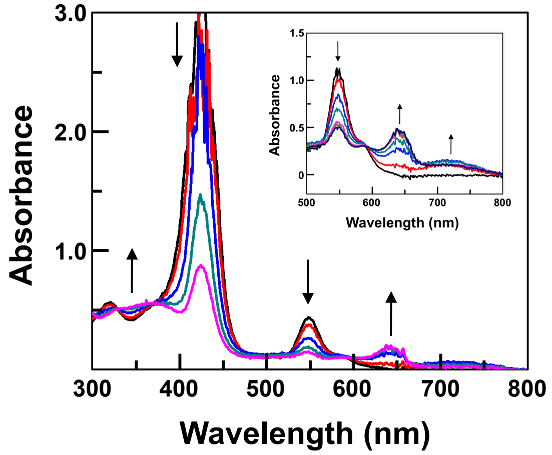

Figure S12. UV-vis spectrum change of 0.25 mM **1** during electrolysis at 1.40 V in a pH 3.0 phosphate buffer solution with Pt mesh as the working electrode, Pt wire as the counter electrode and Ag/AgCl as the reference electrode. Inset: UV-vis spectrum change of 0.80 mM **1** at the 500-800 nm range during electrolysis.

The absorbance at 425 and 550 nm decrease, while the absorbance at 600-800 nm range increase. This result indicates the porphyrin-centered oxidation.

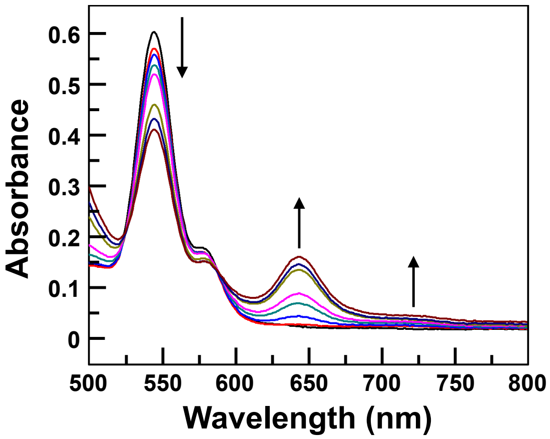

Figure S13. UV-vis spectrum change of **1** at the 500-800 nm range in the propylene carbonate (PC) solution following addition of ceric ammonium nitrate (CAN).

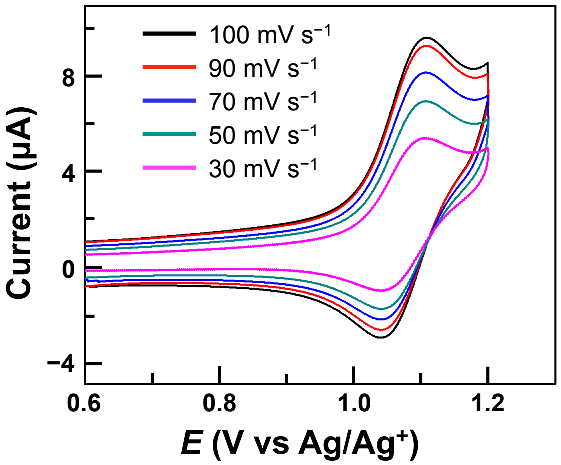

Figure S14. CVs of 0.5 mM **1** in 0.1 M Bu_4NPF_6 propylene carbonate (PC) solution with different scan rates. Conditions: GC working electrode, Ag/Ag^+ reference electrode, 20 °C.

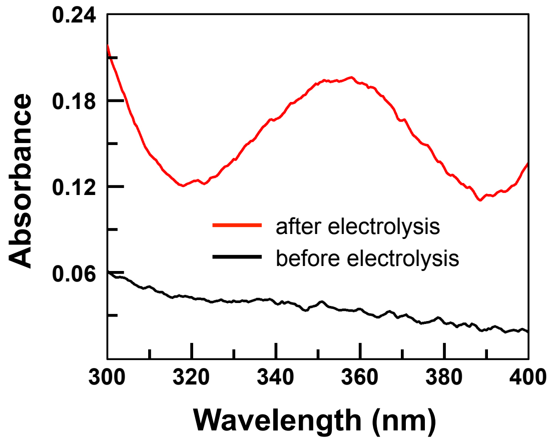

Figure S15. UV-vis absorption spectra of **1** in a pH 3.0 phosphate buffer solution before (black line) and after (red line) the electrolysis at 1.40 V treated with an excess amount of sodium iodide for H_2O_2 detection. Conditions: GC working electrode, 20 °C.

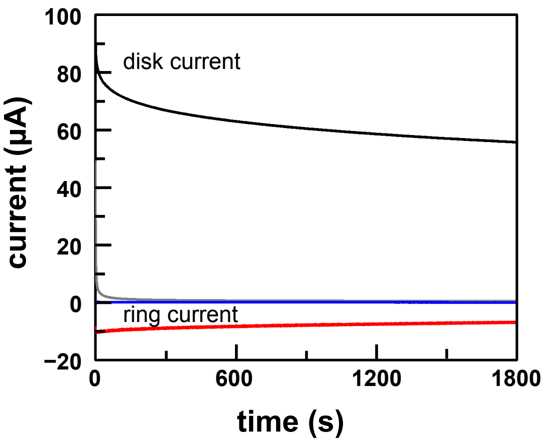

Figure S16. Amperometry with 1.0 mM **1** (black and red) and without (grey and blue) in a pH 3.0 phosphate buffer solution using RRDE at 1600 rpm. Conditions: GC disk electrode at 1.40 V, Pt ring electrode at 0.70 V. This result gives a Faradaic efficiency of 45% for H_2O_2 generation.

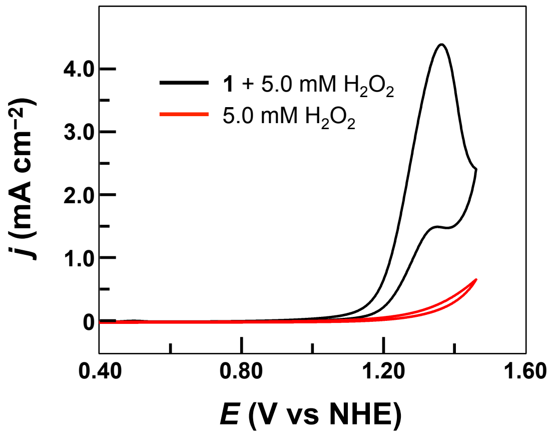

Figure S17. CVs of 5.0 mM H_2O_2 in 0.1 M pH 7.0 phosphate buffer with or without **1** (0.75 mM). Conditions: GC working electrode, scan rate 100 mV s^{-1}, 20 °C.

Table S1. Crystal data and structure refinement parameters for the X-ray structure of **1**.

complex	Cu-TMPyP (**1**)
molecular formula	$C_{48}H_{36}CuF_{12}N_8O_{14.13}S_4$
formula wt. (g mol^{-1})	1370.76
temperature (K)	150(2)
radiation (λ, Å)	0.71073
crystal system	Triclinic
space group	$P\bar{1}$ (#2)
a (Å)	16.1504(11)
b (Å)	16.2726(12)
c (Å)	17.8745(13)
α (°)	99.297(3)
β (°)	112.765(2)
γ (°)	102.854(2)
Volume (Å^3)	4059.7(5)
Z	3
ρ_{calcd} (g cm^{-3})	1.682
μ (mm^{-1})	0.674
F(000)	2082
crystal size (mm^3)	0.30 × 0.30 × 0.30
Theta range	2.23 to 26.41°
reflections collected	73999
indep. reflections	16605 [R(int) = 0.0290]
Completeness	99.5%
goodness-of-fit on F^2	0.997
final R indices	$R_1{}^a = 0.0895$
[R > 2σ (I)]	$wR_2{}^b = 0.2470$
R indices (all data)	$R_1{}^a = 0.0993$<br>$wR_2{}^b = 0.2578$
largest diff. peak and hole (e Å^{-3})	4.418 and −3.902

$^aR_1 = \Sigma||F_o| - |F_c|| / |F_o|$, $^bwR_2 = \{\Sigma[w(F_o^2 - F_c^2)^2] / \Sigma[w(F_o^2)^2]\}^{0.5}$
